# Developing mango powders by foam mat drying technology

**DOI:** 10.1002/fsn3.3397

**Published:** 2023-04-26

**Authors:** Nhi Thi Yen Tran, Thanh Thao Thi Le, Nguyen Huu Nghia, Dang Be Nhu, Long Bao Huynh, Tan Xuan Tung Nguyen, Phong Xuan Huynh, Tan Phat Dao

**Affiliations:** ^1^ Institute of Applied Technology and Sustainable Development Nguyen Tat Thanh University Ho Chi Minh City Vietnam; ^2^ Faculty of Environmental and Food Engineering Nguyen Tat Thanh University Ho Chi Minh City Vietnam; ^3^ Faculty of Chemical Engineering and Food Technology Nong Lam University Ho Chi Minh City Vietnam; ^4^ Faculty of Chemical Engineering Ho Chi Minh City University of Food Industry Ho Chi Minh City Vietnam; ^5^ Center of Water Management and Climate Change, Institute for Environment and Resources Vietnam National University, Ho Chi Minh City (VNU‐HCM) Ho Chi Minh City Vietnam; ^6^ Department of Microbial Biotechnology, Institute of Food and Biotechnology Can Tho University Can Tho City Vietnam

**Keywords:** arabic gum, foam mat drying, maltodextrin, mango powder

## Abstract

Using mango purée from overripe mangoes to produce powders helped to solve agricultural product stagnation. The research investigates the effect of thickening additives, convection drying, and heat pump drying on bioactive compounds such as total phenolic content (TPC), total flavonoid content (TFC), color, and solubility of the final product. The obtained results showed that the mixture (gum arabic and maltodextrin in the ratio 50:50 w/w) at a concentration of 15% gave a good quality powder texture when dried by hot air convection at 55°C with TPC (21.24 ± 1.58 mg GAE/g dry weight [DW]) and TFC (0.34 ± 0.02 mg QE/g DW), respectively. In addition, the product has a high solubility of 64.35%, with the highest pass‐through point of 17.11.

## Using mango purée from overripe mangoes to produce powders helped to solve agricultural product stagnation. The research focuses on investigating the effect of thickening additives; convection drying, and heat pump drying on bioactive compounds such as total phenolic content, total flavonoid content, color, and solubility of the final product.

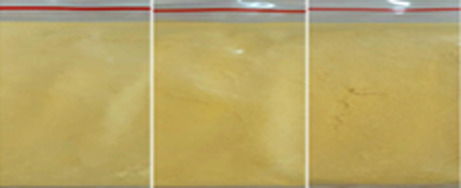

## 1 INTRODUCTION

Mango (*Mangifera indica* L.) originated in Asia, especially from the Indo‐Burmese region, about 4000 years ago (Masibo & He, 2009). Currently, there are more than 1000 varieties of mango in the world, but a few are grown commercially, and there are 69 species grown mainly in the tropics (Yadav & Singh, 2017). Mangoes contain many vital antioxidant compounds such as isoquercitrin, quercetin, fisetin, methyl gallate, gallic acid, and astragalin that help prevent the formation of free radicals, help slow down the aging process, and prevent cancer. Mangoes contain high levels of vitamin C, pectin, and fiber, reduce serum cholesterol levels, help to improve blood lipid disorders, brighten eyes, strengthen the immune system, support the excretory system, and promote health (Huong et al., 2022; Maldonado‐Celis et al., 2019).

Foam mat drying is an alternative to some technologies, such as spray drying and freezing drying. The liquid is whipped to form a stable foam and heat‐dehydrated. The larger surface area of the foam accelerates the drying process to remove moisture quickly from high‐moisture foods. Foam mat drying is converting a product from a liquid to a stable foam that is then air‐dried. Stable air–liquid bubbles are the primary condition for successful foam mat drying. Proteins, gums, and various emulsifiers (e.g., glycerol monostearate, propylene glycerol monostearate, carboxymethyl cellulose, and trichlorophosphate) are used as foaming agents. Drying is carried out at relatively low temperatures to form a honeycomb‐shaped sheet or sheet, which decays to produce a free‐flowing powder. The larger surface area exposed to the drying air is the main cause of accelerated moisture loss (Quenzer & Burns, 1977). However, capillary diffusion is also the main cause of moisture migration inside the product during the drying process of the foam sheet (Sankat & Castaigne, 2004). The advantages of the foam mat drying process include suitability of all juices, quick drying at lower temperatures, preservation of nutritional qualities, ease of reconstitution, and efficiency for powder production fruit juice easy to reconstitute (Ratti & Kudra, 2006). The foam carpet drying process is reported to be significantly cheaper than the vacuum, freeze, and spray drying methods (Kadam et al., 2010). Several recent studies have shown interest in mango processing technology, such as that of Shende and Datta (2020) that have optimized the refractive window drying process of mango cultivars of the Langra variety (Shende & Datta, 2020). Kadam et al. (2010) studied the process of foaming to create mango powder, by whipping the mango juice mixture with the addition of liquid milk (concentrations of 0%, 10%, 15%, 20%, and 25%), 8.5% of nonfat condensed milk (SNF), 4.5% of fat, 0.6% of minerals, and 3.3% of protein, with a hand blender for 3 min until a homogeneous foam is obtained. Then, the foam was poured into a steel tray, was spread, 3 mm thick, and dried at 65°C. After drying, it was scraped and ground into powder. The values of total sugar, ascorbic acid, total carotene content, and minerals retained high during processing and microbial content within the allowable thresholds (Kadam et al., 2010). Dereje and Abera (2020) conducted a study on the effects of pretreatment and drying methods on the quality of dried mango slices. The survey results show that fluidized bed drying and freeze drying combined with pretreatment methods help retain the color and content of antioxidant compounds the best (Dereje & Abera, 2020).

The Mekong Delta is the largest mango growing area in Vietnam, with 55% of the area spreading across provinces such as Dong Thap (about 9600 ha, output of more than 90,000 tons/year), Tien Giang, Can Tho, and Ben Tre, with many famous mango varieties: Hoa Loc mango, Cat Chu mango, Tuong mango, Thom mango, and Tu Quy mango which brings economic benefits to mango growers and Vietnam's agricultural economy (Phú & Khương, 2018). Among internationally traded tropical fruits, mangoes are second to bananas in quantity and fifth in total production among significant fruit trees worldwide. Mango production in the world is estimated at more than 26 million tons. India ranks first among the world's mango‐producing countries, accounting for 54.2% of all mangoes produced worldwide and it is the most essential fruit tree in India, with over a thousand varieties known so far. Prominent mango exporting countries include China, Thailand, Indonesia, Philippines, Pakistan, and Mexico (Jahurul et al., 2015). The development of mango in both quantity and quality is significant for the agricultural economy of Vietnam. However, at present, the mango growing provinces have not yet adequately assessed the value of mangoes. The complex problem of the country's agriculture is “good harvest, loss of value”, reducing the income of mango growers. Tu Quy mango tree is considered one of the main crops of farmers in coastal communes such as Thanh Phong, Thanh Hai, and Giao Thanh (Thanh Phu district) with more than 700 households participating in planting, with a total area of over 400 ha, the average yield is 30–40 tons/ha, the average income is from 300 to 400 million/ha/year, and there is a tendency to increase the cultivated area. According to Agriculture Newspaper, Tu Quy mango is a large fruit, with harvested weight from 0.5 to 1 kg/fruit. Evergreen mango, when ripe, has a yellowish‐green color, the fruit flesh also turns pale yellow (not as dark as sand mango) and it is one of the fruits with sudden respiratory properties.

From the above issues, to properly utilize and solve the problem of unsold ripe mangoes, the research aims to use Tu Quy mango purée to develop mango powder products applying foam mat drying technology to storage of compounds with antioxidant activity in mango.

## 2 MATERIALS AND METHODS

### 2.1 Preparation of material

**FIGURE 1:**
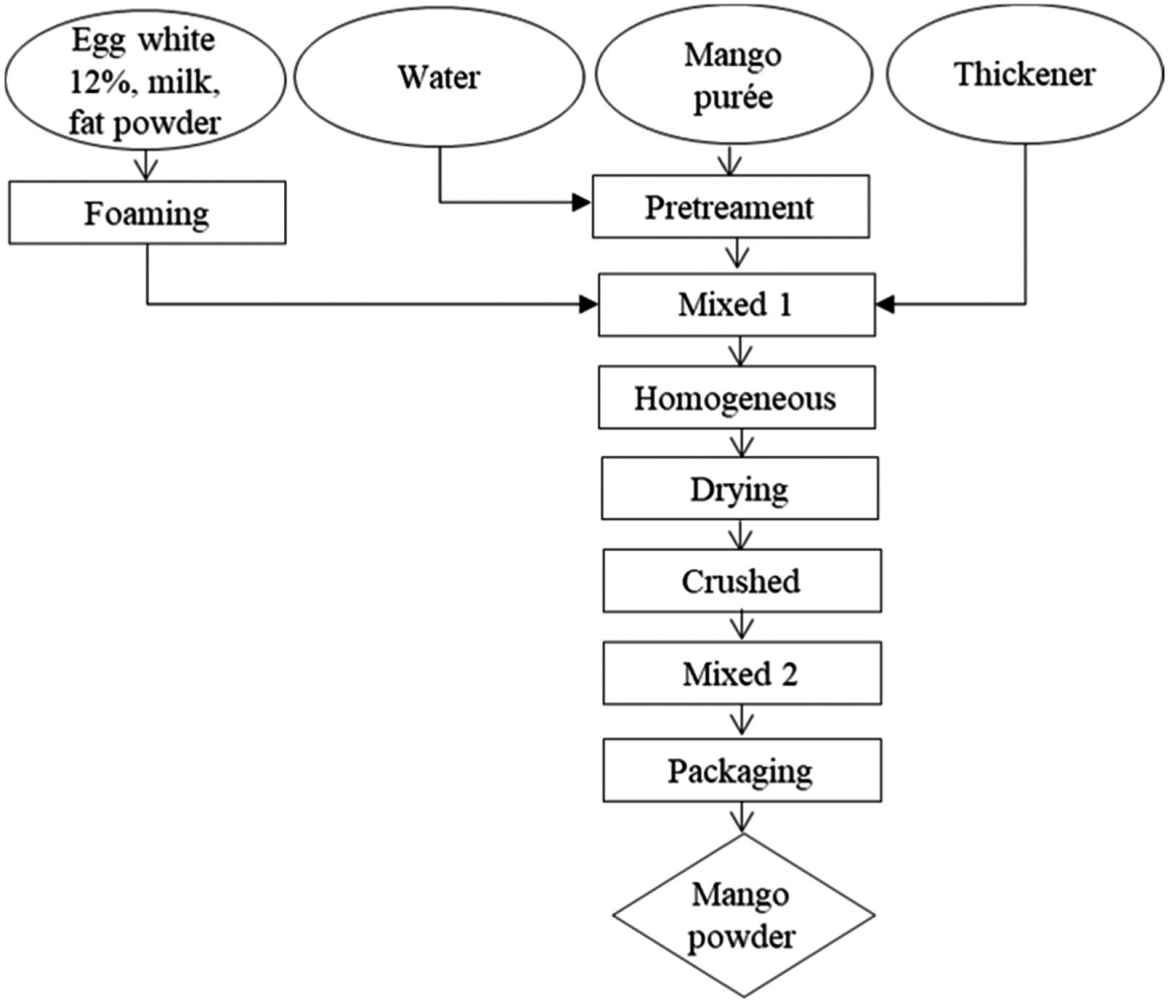
Technological process of producing mango powder.

Mango powders processing has been based on model of Kadam et al. (2010) and modified in Figure 1. Overripe mangoes are peeled and seeded, using the pulp to purée into purée. Then, the mango purée was pretreated at 90°C for 150 s to inactivate the browning enzyme. At the same time, 12% of egg white, 10% of milk, and 8.5% of fat powder were added and whipped with a whisk for 3 min. Next, the whole mixture was mixed with the mango purée, the solution was spread on the drying tray with a thickness of 2 mm, and dried at different temperature ranges according to arrangement until the moisture content reaches less than 3%. The mixture was ground finely, blended with thickener, and the product was packaged.

### 2.2 Chemicals and agent

Folin–Ciocalteu and Gallic acid were purchased from Sigma‐Aldrich; dichlorophenolindophenol (DCPIP) (purity 99.7%), HCl (purity 36.5%), and Na_2_CO_3_ (purity 99.5%) from China; maltodextrin (D12) and arabic gum were purchased from Alland & Robert; eggs, milk, and fat powder were purchased from Co.op Mart supermarket.

### 2.3 Determination of total ascorbic acid

The ascorbic acid content was determined according to the method of AOAC 967.21, which was evaluated on cashews earlier by Dao et al. (2021). First, 1 g of the sample was extracted three times and then titrated to 100 mL with distilled water. Next, 10 mL of sample solution added to 1 mL of 0.04% HCl and titrated with DCPIP. The control sample was carried out in the same way as above, and the analytical sample was diluted with 0.1 g of ascorbic acid up to 100 mL. Finally, both samples were repeated three times and titrated from colorless solution to pale pink within 30 s and the volume of DCPIP solution was recorded.

Total ascorbic acid (TAA) content by dry weight (DW) is calculated as follows:

(1) $$\mathrm{TAA}\;\left(\mathrm{mg}/g\;\mathrm{dry}\;\text{weight}\right)=\frac{\left\{\left(V_{1}-0.05\right)\times V_{2}\times 10\times m_{1}\times \mathrm{df}\right\}\times m_{2}}{10\times m_{2}\times \left(V_{3}-0.05\right)\times m_{3}}$$

where *V*_1_ is the average DCPIP volume of the sample, mL; *V*_2_ is the volume of the container of the extracted sample, mL; *m*_1_ is the common standard mass of ascorbic acid, g; df is the sample dilution factor; *V*_3_ is the DCPIP volume of ascorbic acid standard, mL; and *m*_3_ is the sample mass according to the dry concentration, g.

### 2.4 Determination of total phenolic content

Polyphenol content was determined by Folin–Ciocalteu colorimetric method (Nguyen et al., 2019). First, 1 g of the sample was extracted three times and titrated to 100 mL with distilled water. Next, the collected filtrate (0.5 mL) was transferred into a dark tube, followed by the addition of 2‐mL Folin–Ciocalteu reagent (diluted 10 times with distilled water) and 2.5‐mL sodium carbonate solution (20% w/v). The mixture was then incubated in the dark for 1 h before being measured photometrically at an absorption wavelength of 765 nm.

Total polyphenol content (TPC) by DW is calculated as follows:

(2) $$\mathrm{TPC}\;\left(\mathrm{mg}/g\;\mathrm{DW}\right)=\frac{V_{1}\times \mathrm{df}\times C_{1}\times 0.1\times V_{2}}{V_{3}\times \left(100-h\right)}$$

where *V*_1_ is the volume of the cuvet, mL; *V*_2_ is the volume of the container of the extracted sample, mL; *V*_3_ is the extract volume of fresh sample, mL; *C*_1_ is the polyphenol concentration measured from Ultraviolet–Vis, μg/mL; and *h* is the moisture in the sample, %.

### 2.5 Dissolution ability

The solubility was determined according to the method of Anderson (1982). Place 2.5 g powder and 30 mL distilled water in a 50 mL centrifuge tube. Shake the sample mixture vigorously for 1 min and incubate in a water bath at 37°C for 30 min, then centrifuge at 2785 *g* for 20 min. The supernatant powder was collected, transferred to a petri dish, and dried at 105°C to constant weight. The solubility (WAI) was calculated according to the following formula:

(3) $$\mathrm{WAI}\;\left(\%\right)=\frac{W_{s}\times 100}{W_{i}\;}$$

where *W*_s_ is the mass after drying and *W*_i_ is the initial sample volume.

### 2.6 Colorimetric method

The CIE Lab* color space is a reference color option based on three values L*, a*, and b* (Torres et al., 2011). Brightness was measured through a Chroma Scanner Colorimeter (model NR60CP). Results are displayed as numbers through L* (brightness ranges from 0 to 100), a* (green to red), and b* (blue to yellow) values.

### 2.7 Determination of free radical scavenging activity by 1,1‐diphenyl‐2‐picrylhydrazyl

The sample was diluted to the appropriate concentration, and 0.5 mL of the sample was drawn into the test tube. The control sample replaced ethanol (99.5%). Then, 1.5 mL of 1,1‐diphenyl‐2‐picrylhydrazyl (DPPH) solution was drawn into a test tube (corrected OD_517_ nm = 1.1 ± 0.02) and left in the dark for 30 min. The optical absorbance was measured at 517 nm on an Ultraviolet–Vis spectrophotometer. Vitamin C (ascorbic acid) was used as the standard for comparison. The ability to neutralize free radicals by DPPH was calculated as the percentage of milligram equivalent of ascorbic acid in 1 g dry matter compared with the original sample according to the equation *y* = −0.1239*x* + 0.991 (*R* ^2^ = 0.999999).

### 2.8 Determination of free radical scavenging capacity by ABTS

ABTS free radical solution was prepared by adding 10 mL of 7.4 mM ABTS solution to 10 mL of 2.6 mM K_2_S_2_O_8_ solution, incubating in the dark for 24 h, and then diluting with ethanol and conditioning. The absorbance of the solution was adjusted at 734 nm to 1.1 ± 0.02. the sample was diluted to the appropriate concentration range and0.5 mL of the diluted sample was drawn into the test tube. The control sample replaced ethanol (99.5%). 1.5 mL of ABTS solution (OD_734_ nm = 1.1 ± 0.02) was drawn into a test tube and left in the dark for 30 min. The optical absorbance was measured at 734 nm on an Ultraviolet–Vis spectrophotometer. Vitamin C (ascorbic acid) was used as the standard for comparison. The equation *y* = −0.1266914*x* + 0.6672952 (*R* ^2^ coefficient = 0.99936) was used to calculate the percentage of milligram equivalents of ascorbic acid in 1 g dry matter compared with the original sample for ABTS free radical scavenging capacity.

### 2.9 Sensory evaluation method

Implementation method according to TCVN 3215‐79. This standard specifies a method for checking the quality of food products by sensory scoring, applicable to check all the organoleptic criteria or each criterion (color, smell, taste, etc.) of each product and good. This method is based on assessing the sensation that appears according to its type and intensity.

### 2.10 Statistical analysis

The experimental layout was triplicate and data were entered and processed by Excel software. The program Statgraphics Centurion XV.I was used to analyze one‐factor ANOVA at the significance of the level of *p* < .05.

## 3 RESULTS AND DISCUSSION

### 3.1 Effect of mixing process on the quality of mango powder product

**FIGURE 2:**
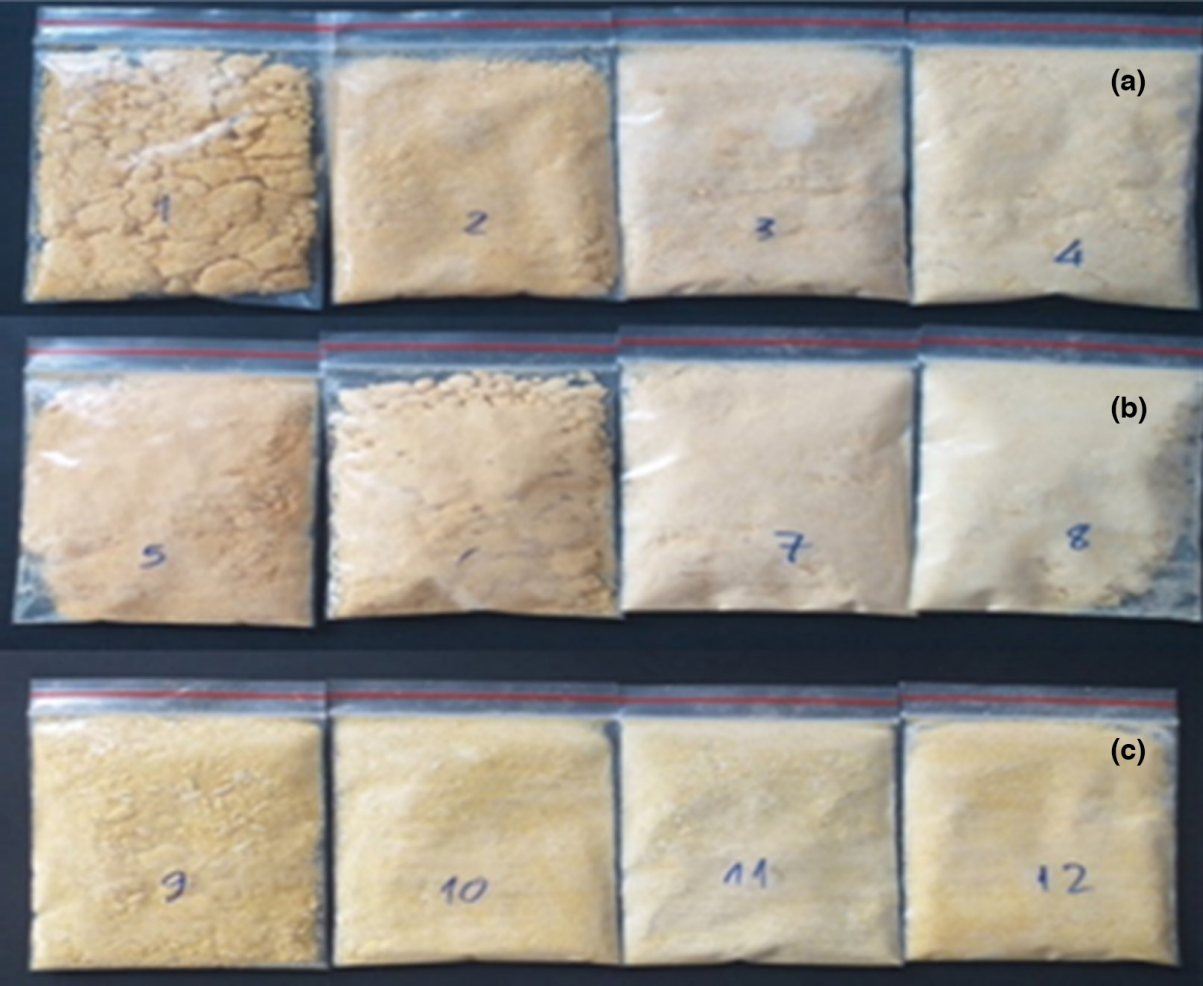
Mixing different carrier concentrations in mango pulp (a) gum arabic; (b) maltodextrin; (c) mixture (gum arabic and maltodextrin 1:1 w/w).

Color is an important quality indicator that affects consumer acceptance and the market potential of a product. The color parameters (L*, a*, b*, and ΔE*) and the surface change of mango pulp are presented in Table 1 and Figure 2, respectively. L* (darkness/brightness), a* (greenness/redness), and b* (blueness/yellowness) are used to calculate ΔE*. All color values of the carrier samples were increased compared with the mango purée sample. This was primarily due to the relatively high drying time and temperature leading to the color deterioration (Nhi et al., 2020; Zhao et al., 2015); however, the color is not too different from the mango purée.

**TABLE 1:** Effect of carrier concentration on ∆*E*. Solubility and bioactive compounds in mango pulp.

Evaluation criteria sample	Concentration (%)	∆E*	Solubility (%)	Total phenolic content (mg/gGAE dry weight [DW])	Total ascorbic acid (mg/g DW)	Total flavonoid content (mg/g DW)	1,1‐diphenyl‐2‐picrylhydrazyl (%)	ABTS (%)
Control	‐	‐	‐	188.99 ± 8.42	0.64 ± 0	4.16 ± 0.03	29.82 ± 0.04	0.73 ± 0.01
Arabic gum	5	22.19 ± 0.27	50.67	11.82 ± 0.75^a1^	0.08 ± 0^a1^	0.5 ± 0.01^a1^	3.84 ± 0.01^a1^	0.58 ± 0.05^a1^
	10	23.18 ± 0.9	60	11.56 ± 0.06^a1^	0.15 ± 0.04^a1^	0.35 ± 0.03^b1^	0.74 ± 0.03^b1^	0.09 ± 0^b1^
	15	27.48 ± 0.15	64	11.06 ± 0.03^a1^	0.1 ± 0.02^a1^	0.38 ± 0.01^c1^	1.32 ± 0.01^c1^	0.3 ± 0.02^c1^
	20	26.67 ± 0.57	64	10.32 ± 0.75^a1^	0.1 ± 0.02^a1^	0.24 ± 0.01^d1^	2.81 ± 0.02^d1^	0.06 ± 0.01^c1^
Maltodextrin	5	26.02 ± 1.03	40	19.69 ± 0.35^a2^	0.11 ± 0^a2^	0.44 ± 0.03^a2^	5.63 ± 0.01^a2^	0.3 ± 0.01^a2^
	10	26.35 ± 3.11	44	24.24 ± 1.58^a2^	0.12 ± 0.04^a2^	0.34 ± 0.02^b2^	3.56 ± 0.01^b2^	0.13 ± 0^b2^
	15	27.8 ± 1.77	60	18.67 ± 1.15^a2^	0.07 ± 0.02^a2^	0.32 ± 0.01^b2^	2.87 ± 0.04^c2^	0.05 ± 0^c2^
	20	29.51 ± 1.24	73.33	18.47 ± 0.56^a2^	0.07 ± 0.02^a2^	0.25 ± 0.01^c2^	2.45 ± 0.02^d2^	0.04 ± 0^c2^
Mixed	5	25.31 ± 0.62	66.67	16.28 ± 0.33^a3^	0.06 ± 0.04^a3^	0.42 ± 0.01^a3^	4.22 ± 0.02^a3^	0.27 ± 0.02^a3^
	10	26.47 ± 1.87	69.33	19.1 ± 1.13^a3^	0.11 ± 0.04^a3^	0.36 ± 0^b3^	3.82 ± 0.01^b3^	0.21 ± 0.02^b3^
	15	30.61 ± 1.41	74.67	15.88 ± 1.21^a3^	0.07 ± 0.02^a3^	0.46 ± 0.01^c3^	3.77 ± 0.01^c3^	0.17 ± 0^c3^
	20	28.72 ± 1.92	82.67	3.65 ± 0.11^a3^	0.09 ± 0.02^a3^	0.18 ± 0.02^d3^	2.36 ± 0.02^d3^	0.09 ± 0^d3^

*Note*: (a–d) difference between carrier concentrations after ANOVA treatment; (1–3) distinguish thickeners in the same row.

The results in Table 1 and Figure 2 show that the color difference is significant when increasing the carrier concentration, as indicated by the ∆E* value (the more significant the E* value, the greater the difference with the untreated sample.), and is partly due to the effects of drying temperature and the presence of carriers. However, they can act as a color fastness aid. In general, the color of the samples did not change too much. An increase in L* values indicates a decrease in the white color intensity due to yellow pigments (Manolopoulou & Varzakas, 2016). More prominently, the mixed gum arabic and maltodextrin sample showed that the solubility was 74.67%, and the color was moderate, not too much different from the pure mango powder sample. This may be because gum arabic has a higher viscosity and can form a more stable system than maltodextrin (Chen et al., 1998; Nhan et al., 2020).

The mixture (gum arabic and maltodextrin in the ratio 50:50 w/w) was selected at a concentration of 15% for further experiments because of its good organoleptic qualities, no clumping, bright colors, aroma, and active activities; biology is less affected.

### 3.2 Effect of foam mat drying on the quality of mango pulp

**FIGURE 3:**
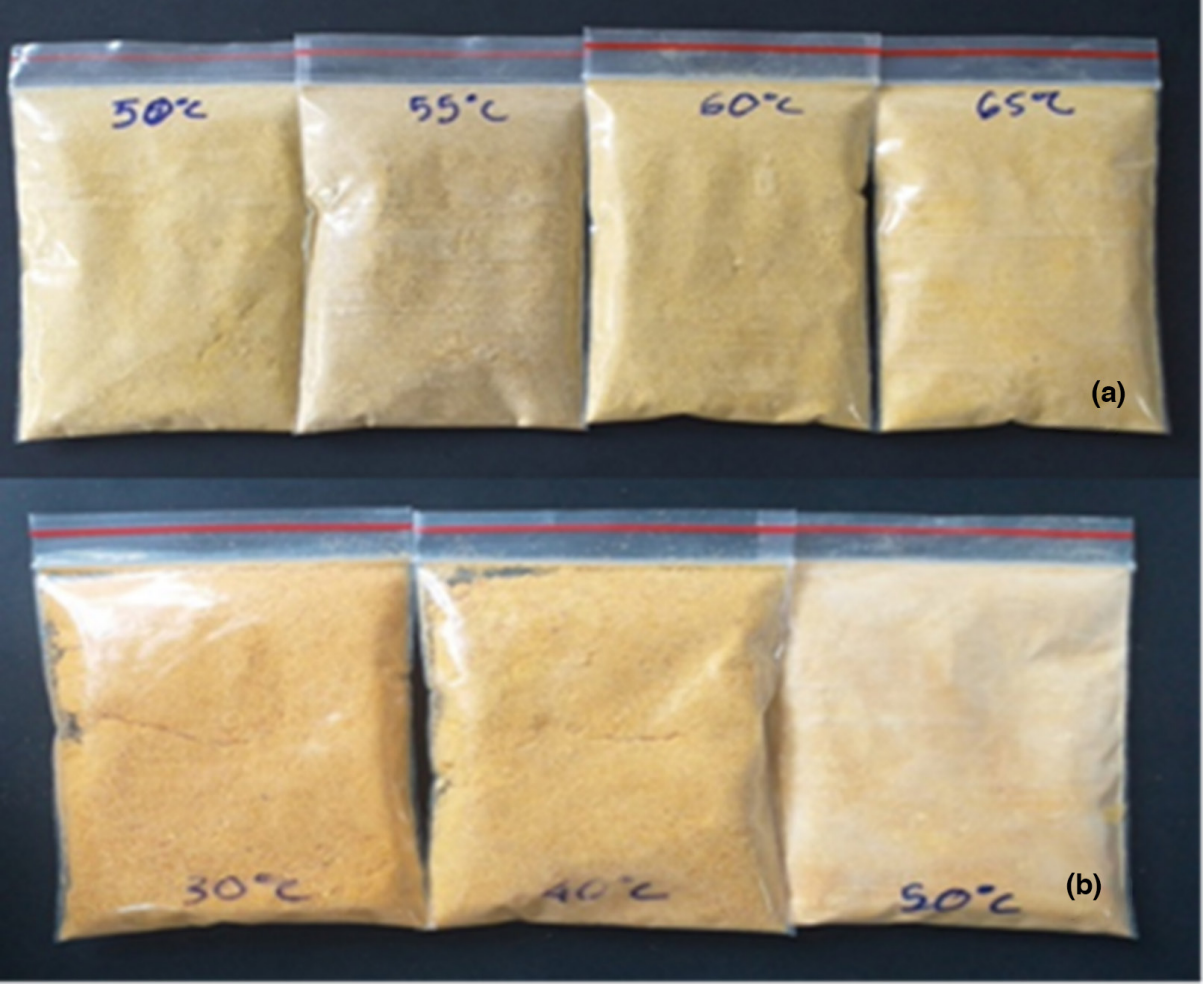
Description of powder at different convection and heat pump drying temperatures (a) convection drying (50‐55‐60‐65°C); (b) heat pump drying (30‐40‐50°C).

The solubility of mango powder given in Table 2 and colors in Figure 3 showed that with increasing convection drying temperature, the solubility decreased to 76.99%. This trend is opposite with heat pump drying technology, specifically, the solubility increased from 73.09% to 82.56% when increasing the drying temperature from 30 to 50°C. However, this value has reached 98.84%, the highest when convection drying at 55°C. In addition, the color difference values have shown that heat pump drying has a possible result in giving a bright yellow color to mango pulp; however, some bioactive compounds are not retained much at low concentrations. Specifically, the TPC value from 188.99 ± 8.42 mg/g DW was reduced to 12 ± 0.04 mg/g DW (50°C), giving the TAA value (from 0.64 mg/g DW) remaining 0.08 ± 0.02 mg/g DW, total flavonoid content (TFC) remaining 0.82 mg/g DW; DPPH remaining 1.58 ± 0.08 mg/g DW compared to the original 29.82 ± 0.04 mg/g DW. This may be due to the low drying temperature and continuous drying for a long time, leading to long exposure to heat and environment, so the bioactive compounds are decomposed (Nhi et al., 2022; Ortiz et al., 2013).

**TABLE 2:** Effect of foam mat drying on color deviation, solubility, and bioactive compounds.

Evaluation criteria sample	Temp. (°C)	∆E*	Solubility (%)	Total phenolic content (mg GAE/g dry weight [DW])	Total ascorbic acid (mg/g DW)	Total flavonoid content (mg QE/g DW)	1,1‐diphenyl‐2‐picrylhydrazyl (mg/g DW)	ABTS (mg/g DW)
Control	‐	‐	‐	188.99 ± 8.42^a1^	0.64 ± 0^a2^	4.16 ± 0.03^a3^	29.82 ± 0.04^a4^	0.73 ± 0.01^a5^
Convection drying	50	26.02 ± 1.03	89.2	19.69 ± 0.35^c1^	0.11 ± 0^c2^	0.44 ± 0.03^cd3^	5.63 ± 0.01^b4^	0.3 ± 0.01^c5^
	55	26.35 ± 3.11	98.84	21.24 ± 1.58^bc1^	0.12 ± 0.04^c2^	0.34 ± 0.02^d3^	3.56 ± 0.01^c4^	0.13 ± 0^d5^
	60	27.8 ± 1.77	82.87	18.67 ± 1.15^cd1^	0.07 ± 0.02^d2^	0.32 ± 0.01^d3^	2.87 ± 0.04^d4^	0.05 ± 0 ^e5^
	65	29.51 ± 1.24	76.99	18.47 ± 0.56^cd1^	0.08 ± 0.02^d2^	0.25 ± 0.01^e3^	2.45 ± 0.02^e4^	0.04 ± 0^e5^
Heat pump drying	30	17.48 ± 3.92	73.09	17.16 ± 0.03^d1^	0.64 ± 0^b2^	0.54 ± 0^c3^	1.14 ± 0.02^f4^	0.12 ± 0^d5^
	40	17.91 ± 1.76	80	16.25 ± 0.02^d1^	0.1 ± 0.01^d2^	0.49 ± 0.02^cd3^	1.32 ± 0.01^fg4^	0.18 ± 0.01^d5^
	50	18.43 ± 0.27	82.56	12 ± 0.04^e1^	0.08 ± 0.02^d2^	0.82 ± 0^b3^	1.58 ± 0.08^f4^	0.39 ± 0^b5^

*Note*: (a–d) the difference between the postdrying temperatures; (1–5) distinguish between columns.

Selecting convection drying technology at 55°C gave positive results in terms of color and high solubility, in addition to helping to preserve the highest biologically active ingredients.

### 3.3 Investigation of the influence of the mixing process on the sensory quality

#### 3.3.1 Effect of thickener ratio on the product's taste

The results in Tables 3 and 4 showed that the solubility of mango purée samples in the presence of carriers (tapioca starch and cornstarch) was higher than that of samples without carriers. However, the sample containing tapioca starch had better solubility than the sample containing cornstarch. The reason is that tapioca starch has better water‐holding capacity than cornstarch (Hirai & Odani, 1994). The taste of the cornstarch sample was sourer than that of the tapioca sample (Ali et al., 2009). In addition, because the smell of cornstarch was partly related to the essential smell of mango powder, we have selected tapioca starch mixed with a concentration of 9% for good solubility and product appearance.

**TABLE 3:** Description of mango powder properties.

Thickener	Concentration %	Texture	Color	Odor	Taste
Tapioca	5	Flour smooth	Light yellow	Odorous	Sweet, slightly sour
	7	Flour smooth	Light yellow	Odorous	Sweet, slightly sour
	9	Flour smooth	Light yellow	Odorous	Sweet, slightly sour
Corn starch	5	Flour smooth	Light yellow with orange stains	Odorous	Sweet, slightly sour
	7	Flour smooth	Light yellow with orange stains	Odorous	Sweet, sour
	9	Flour smooth	Light yellow with orange stains	Odorous	Sweet, sour

**TABLE 4:** Effect of mixing agent concentration on the solubility of mango powder.

	Purée mango	Tapioca			Corn starch		
Concentration (%)	‐	5	7	9	5	7	9
The solubility (%)	40	62.49	63.04	64.35	59.11	60.83	62.11

#### 3.3.2 Effect of the proportion of flavoring additives on the sensory properties of the product

**FIGURE 4:**
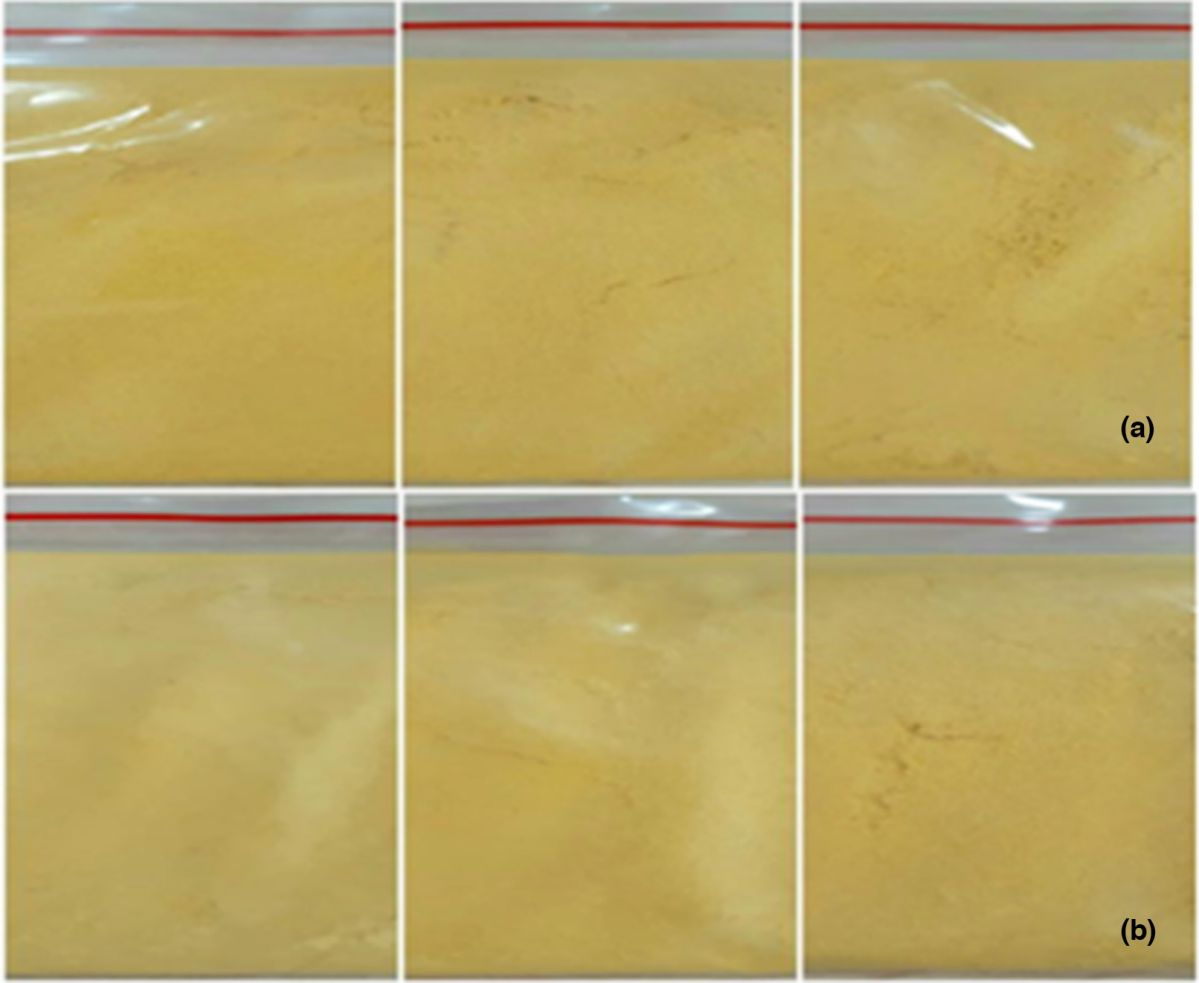
Mixing concentrations of citric acid and sugar in different proportions (a) citric acid concentration (0.005%‐0.010%‐0.015%); (b) concentration of powdered sugar (2%‐4%‐6%).

Solubility, flavor enhancer, and the sensory scores of mango powder have been illustrated in Tables 5 and 6 and Figure 4. Sensory scores when mixing citric acid have shown that the sample mixed with 0.015% has the lowest total score (14.01), in which there is a score of taste assessment giving poor results (2.87 ± 0.83) while the influence coefficient of this criterion accounts for 1.8/4 according to TCVN 3215‐79. On the other hand, the highest total score value was 15.12 at the mixing concentration of 0.005% citric acid. It is likely that, because of the high total acid availability in the quartet mango purée, a low addition rate is more acceptable perceptually. Therefore, the concentration of 0.005% citric acid was selected for mixing, and sensory evaluation was carried out on the sugar mixing ratio. The results showed that, at 4% sugar compared to the starting material, it was accepted and had little effect on the appearance, in which the structural index score reached 4.27 ± 0.96; color score of 4.4 ± 0.74, taste score of 4.2 ± 0.94, favorite product score of 4.27 ± 0.88, and the highest weighted total acceptance score at 17, 11, this value is 11.2 more elevated than the Vietnam Standard on the acceptability of products to be circulated in the market.

**TABLE 5:** Effect of flavoring additives on the solubility of mango powder.

	Mango powder	Citric acid			Sugar		
Concentration (%)	‐	0.005	0.01	0.015	2	4	6
The solubility (%)	64.35	64.53	64.42	64.11	65.12	65.86	66.23

**TABLE 6:** Effects of flavoring additives on the organoleptic taste of mango powder.

Sample	Concentration (%)	Texture (1)	Color (1.2)	Flavor (1.8)	Favorite	Overall score
Citric acid	0.005	3.8 ± 0.77	4.33 ± 0.72	3.4 ± 0.83	3.47 ± 0.74	15.12
	0.01	3.6 ± 0.83	4.0 ± 0.85	3.53 ± 0.64	3.6 ± 0.83	14.76
	0.015	3.73 ± 0.7	4.27 ± 0.7	2.87 ± 0.83	3.2 ± 0.94	14.01
Selected citric acid concentration 0.005%						
Sugar	2	4.13 ± 0.99	4.27 ± 0.8	4.07 ± 0.96	4.27 ± 0.7	16.57
	4	4.27 ± 0.96	4.4 ± 0.74	4.2 ± 0.94	4.27 ± 0.88	17.11
	6	4.00 ± 1.00	3.93 ± 0.80	3.60 ± 0.83	3.60 ± 0.91	15.20

## 4 CONCLUSION

In general, using auxiliaries in the drying process gave the product a high sensory score. From the obtained results, when using the convection drying method with foaming at 55°C, the drying time is shortened, with little effect on the color and the excellent appearance. In particular, the 15% arabic malto‐gum mixture was selected for good organoleptic performance, the mango pulp was not clumped, the color was bright (with high similarity with the unprocessed purée sample), and the aroma of the fruit was preserved. The mango is naturally ripe and the sweetness is slightly sour. We successfully built a process to produce mango powder from sponge drying technology by convection drying with high sensory value and retaining biologically active compounds. From the data and color, it has been shown that the mixture containing gum arabic has better color retention, but the foam strength is not high. Therefore, a mixture containing a variety of gum and malto was selected for further studies so that the mango powder has the best organoleptic properties, foam stability, and solubility. The quality of mango powder products can be improved by adding functional compounds in upcoming studies.

## AUTHOR CONTRIBUTIONS

**Nhi Thi Yen Tran:** Data curation (equal); formal analysis (equal); methodology (equal); writing – original draft (equal); writing – review and editing (equal). **Thanh Thao Thi Le:** Data curation (equal); investigation (equal). **Nguyen Huu Nghia:** Investigation (equal); methodology (equal). **Dang Be Nhu:** Formal analysis (equal); investigation (equal); methodology (equal). **Long Bao Huynh:** Formal analysis (equal); investigation (equal); methodology (equal). **Tan Xuan Tung Nguyen:** Formal analysis (equal); investigation (equal); methodology (equal); validation (equal). **Phong Xuan Huynh:** Data curation (equal); investigation (equal); methodology (equal); supervision (equal). **Tan Phat Dao:** Formal analysis (equal); investigation (equal); methodology (equal); supervision (equal).

## FUNDING INFORMATION

This research received no specific grant from any funding source.

## CONFLICT OF INTEREST STATEMENT

The authors declare that they have no conflict of interest.

## Data Availability

The data that support the findings of this study are available on request from the corresponding author.
